# Metabolic Effects of Replacing Sugar-Sweetened Beverages with Artificially-Sweetened Beverages in Overweight Subjects with or without Hepatic Steatosis: A Randomized Control Clinical Trial

**DOI:** 10.3390/nu9030202

**Published:** 2017-02-27

**Authors:** Vanessa Campos, Camille Despland, Vaclav Brandejsky, Roland Kreis, Philippe Schneiter, Chris Boesch, Luc Tappy

**Affiliations:** 1 Department of Physiology, Faculty of Biology and Medicine, University of Lausanne, 1005 Lausanne, Switzerland; campos.vanessacaroline@gmail.com (V.C.); camdespland@hotmail.com (C.D.); philippe.schneiter@unil.ch (P.S.); 2 Department of Clinical Research, University Bern and Institute of Diagnostic Interventional and Pediatric Radiology, University Hospital Bern, 3010 Bern, Switzerland; vbrandejsky@gmail.com (V.B.); roland.kreis@insel.ch (R.K.); chris.boesch@insel.ch (C.B.); 3 Graduate School for Cellular and Biomedical Sciences, University Bern, 3012 Bern, Switzerland

**Keywords:** intrahepatocellular lipid concentration, cardiovascular risk factors, fructose, hypertriglyceridemia

## Abstract

Objective: Addition of fructose to the diet of normal weight and overweight subjects can increase postprandial plasma triglyceride and uric acid concentration. We, therefore, assessed whether replacing sugar-sweetened beverages (SSB) with artificially-sweetened beverages (ASB) in the diet of overweight and obese subjects would decrease these parameters. Methods: Twenty-six participants of the REDUCS study, which assessed the effects of replacing SSB by ASB over 12 weeks on intra-hepatocellular lipid concentration, were included in this sub-analysis. All were studied after a four-week run-in period during which they consumed their usual diet and SSBs, and after a 12-week intervention in which they were randomly assigned to replace their SSBs with ASBs (ASB arm) or to continue their usual diet and SSBs (control arm, CTRL). At the end of run-in (week 4) and again at the end of intervention (week 16), they took part in an 8.5 h metabolic investigation during which their plasma glucose, insulin, glucagon, lactate, triglyceride (TG), non-esterified fatty acids (NEFA), and uric acid concentrations were measured over a 30 min fasting period (−30–0 min), then every 2 h over 480 min. with ingestion of standard breakfast at time 0 min and a standard lunch at time 240 min. Breakfast and lunch were consumed together with a 3.3 dL SSB at week 4 and with either an ASB (ASB arm) or a SSB (CTRL arm) at week 16. After analyzing the whole group, a secondary analysis was performed on 14 subjects with hepatic steatosis (seven randomized to ASB, seven to CTRL) and 12 subjects without hepatic steatosis (six randomized to ASB and six to CTRL). Results: Ingestion of meals increased plasma glucose, insulin, glucagon, lactate, and TG concentrations and decreased NEFA concentrations, but with no significant difference of integrated postprandial responses between week 4 and week 16 in both ASB and CTRL, except for a slightly decreased glucagon response in ASB. There was, however, no significant postprandial increase in uric acid concentration in both arms. In the secondary analysis, replacing SSBs with ASBs did not significantly change postprandial TG and uric acid concentrations irrespective of the presence or not of hepatic steatosis, Conclusions: In overweight, high SSB consumers, replacing SSBs with ASBs during 12 weeks did not significantly alter post-prandial TG and uric acid concentration, in spite of the lower energy and fructose content of the meals. These effects were globally the same in subjects without and with hepatic steatosis.

## 1. Introduction

There is presently much concern that high fructose consumption may play a causal role in the development of metabolic and cardiovascular diseases [1,2,3,4]. Furthermore, the unique propensity of fructose to stimulate hepatic de novo lipogenesis (DNL) has made it a prime suspect for the development of non-alcoholic fatty liver disease (NAFLD), although direct evidence remains limited [5,6]. The REDUCS study is a small controlled randomized clinical trial assessing the effects of replacing sugar-sweetened beverages (SSBs) by non-caloric, artificially-sweetened beverages (ASBs) on intra-hepatocellular lipid concentrations (IHCL) in high-SSB consumers with overweight or obesity. Replacing SSB with ASB was the sole intervention, and, although subjects were advised not to change their usual diet, food intake was otherwise left ad libitum. We have reported elsewhere that ASB significantly reduced IHCL compared to control [7]. There was, however, no significant change in body weight, nor on any of the other fasting metabolic parameters. In this setting, we hypothesized that a reduction in IHCL was possibly an early marker of negative energy balance.

In addition to promoting excess energy intake and body weight gain, several investigators have raised concern that fructose-induced DNL may be responsible for dyslipidemia (mainly fasting and postprandial hypertriglyceridemia) [8], tissue lipotoxicity [4], hyperuricemia, and insulin resistance [9] and, hence, that dietary fructose may exert adverse health effects even in the absence of an excess energy intake [10,11]. A secondary outcome of the REDUCS study was to evaluate whether replacing SSBs with ASBs would have beneficial effects on postprandial metabolic homeostasis and, more specifically, on triglyceride and uric acid concentrations. To this purpose, we monitored the plasma concentration of various hormones and metabolites over an 8.5 h period during which participants received one standardized breakfast and one standardized lunch, together with one SSB serving at the end of a four-week run-in period, and either one SSB or one ASB serving according to randomization at the end of the subsequent 12-week intervention. 

It has also been suggested that dietary fructose may exert larger detrimental effects in insulin-resistant than in insulin-sensitive subjects [12], suggesting a potential synergic effect. Since NAFLD is strongly associated with insulin resistance [13], and since IHCL have been proposed to be even more closely associated with an increased cardiometabolic risk than visceral body fat mass, we also ran a secondary analysis to assess the effects of replacing SSBs with ASBs in participants with or without hepatic steatosis. 

## 2. Methods

### 2.1. Subjects’ Inclusion

Male and female subjects with BMI > 25 kg/m^2^ and a habitual daily consumption of two or more servings (22 oz or 660 mL per day or more) of SSBs (defined as carbonated soft drinks and sugar sweetened iced tea) were eligible for this study. Criteria of inclusion were: having maintained a stable body weight (less than 4 kg variation) for the past 18 months; low to moderate physical activity (up to four 30 min-sessions of exercise per week); not being presently attempting to lose weight; and having no known disease. Criteria of exclusion were: any current drug treatment (except oral contraceptive agents); recent or planned pregnancy; consumption of alcohol more than 10 g per day; being on a special diet and; having contraindication for magnetic resonance evaluation.

**Figure 1:**
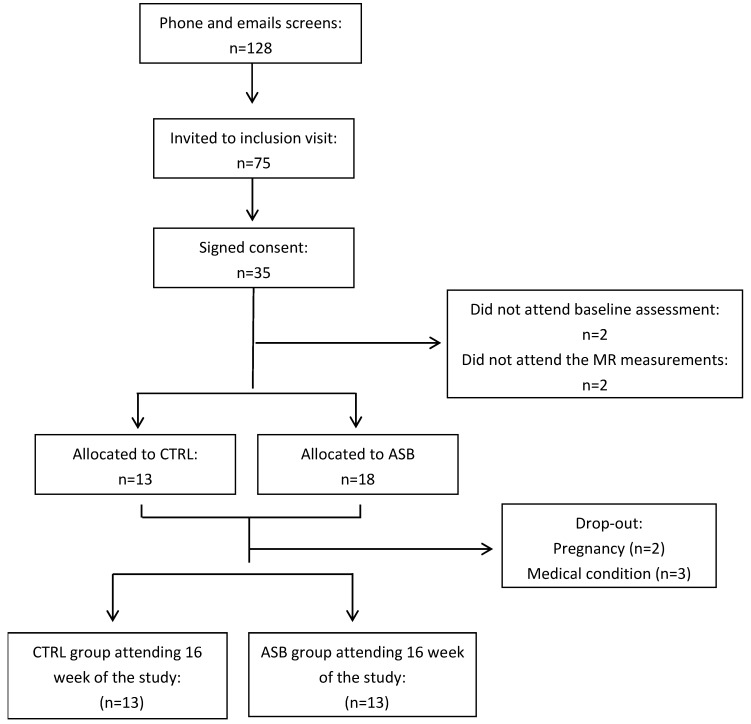
Eligibility, enrollment, randomization, and follow-up of study participants.

Recruitment was done by advertising in the local press and through social networks. The screening, enrollment, randomization, and follow-up of study participants are depicted in Figure 1. Potential participants were initially screened by a phone interview. Seventy-five potential participants were invited for a screening visit including a medical history and a physical examination in order to ensure good physical health. Of 35 subjects found eligible, two quit the study before the first metabolic test, and MRS measurements could not be obtained in another two. The experimental protocol was approved by the Ethical Committee for Human Research of the Canton de Vaud, Switzerland, and registered on clinicaltrials.gov (NCT 01394380). All participants provided an informed, written consent at inclusion. Of the 35 participants initially randomized, five dropped out after the run-in period. The twenty-six subjects (13 in ASB and 13 in CTRL), having completed all metabolic investigations, were included in this secondary analysis. 

After inclusion, all 26 subjects (13 males, 13 females, age range: 20–43 years) entered a four-week run-in period, during which they consumed SSBs and performed their usual daily occupations and physical activity as usual (week 1–week 4). They received every week the number of SSB servings corresponding to their usual weekly consumption, and were asked to return the empty packages on their next weekly visit as a measure of compliance.

During the fourth week, they had to wear an actimeter (DIGI-WALKER SW-2000, Yamax, Japan) and to fill detailed records of their food and beverage intake during two non-consecutive days (working days). For this evaluation, they obtained photographs of each plate they consumed on their mobile phone. A calibration device (pen) was positioned next to the plate to allow subsequent estimation of the quantity of foods present on the plate. At the end of this week, they also had a 60–90 min visit with a nutritionist to verify and discuss their dietary records and to estimate portion sizes. 

On day 27, they reported to the Magnetic Resonance Research Center at the Inselspital Bern, where their intra-hepatocellular lipid (IHCL) concentration and visceral fat volume (VAT) were measured. Between day 27 and 28, they performed a 24-h urine collection. 

On day 28, subjects were asked to come to the Centre of Clinical Research (CRC) at 0700 in the fasting state, and underwent a metabolic test with the ingestion of two test-meals. 

Thereafter, subjects were randomly assigned to consume either SSBs or ASBs for three months. Randomization was stratified by sex. Their food intake and non-sweetened beverages consumption was left ad libitum during this period. The number of either ASB or SSB servings corresponding to their SSB consumption during the run-in period was distributed on a weekly basis, and participants were asked to consume only these beverages, to abstain from any other beverage except for water, tea, or coffee, and to return empty beverage packs at their next visit as a monitoring of compliance 

**Figure 2:**
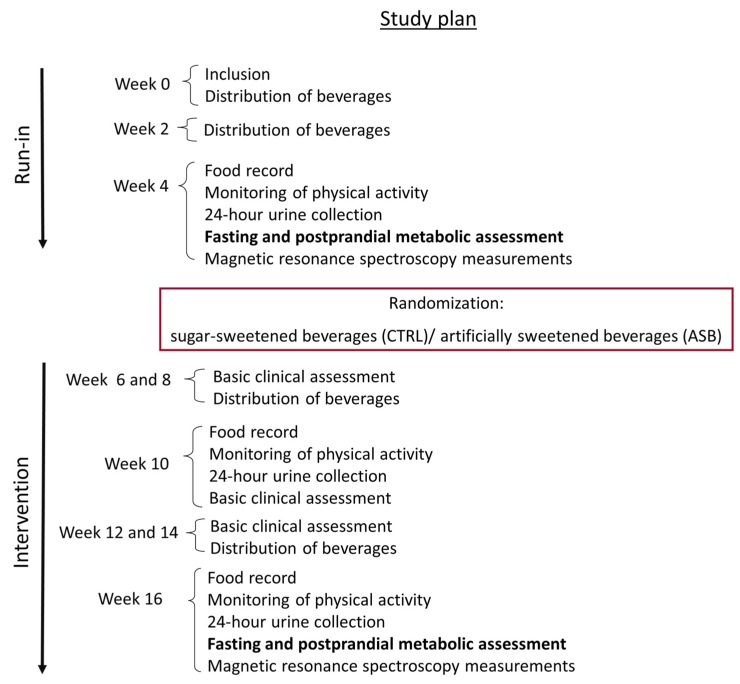
Experimental protocol. Every subject was asked to continue her/his usual diet and physical activity throughout the study; during the initial study, all subjects received SSBs according to their usual consumption (run-in). Thereafter, subjects were randomized to receive either ASB (ASB group) or SSBs (CTRL group) for the next 12 weeks (intervention). Throughout the study, subjects visited the clinical research center every two weeks to collect beverages, return empty cans consumed at home, and for anthropometric measurements and clinical chemistry laboratory (basic clinical assessment). Metabolic investigations were done at week 4 and 16. They also had food their food intake monitored every six weeks.

On weeks 10 and 16, they had their food intake estimated from two-day food records, their physical activity was monitored with an actimeter, and their 24-h urines were collected. These data are, however, not presented here. All of the measurements done at week 4 (end of run-in) were obtained again at week 16 (end of intervention). The study flow chart is shown in Figure 2.

### 2.2. Metabolic Tests

Each subject underwent a detailed metabolic investigation at the end of the run-in period and at the end of the 12-week intervention. For these investigations, subjects reported at about 7 a.m. to the Clinical Research Center of Lausanne University Hospital. They were fasted since 10:00 p.m. the day before, and traveled to the hospital by public transportation, with minimal physical activity involved. At their arrival at the Clinical Research Center, they were asked to void their bladder, and their urine was discarded. Urine was thereafter collected until the end of the test for the determination of the urinary urea nitrogen excretion rate. Subjects were weighed and the body composition was assessed by bio-electrical impedance (Imp. Df. 50; ImpediMed, Pinkenba, Australia). They were then transferred to a bed, where they remained lying until the end of the test. A catheter was inserted in a vein of the right arm, which was subsequently used for blood collection. 

After a 30 min period of baseline measurements, two test-meals, each containing 15% protein, 30% fat, and 55% carbohydrate, were administered at time 0 and time 240 min. Breakfasts and lunches provided 25% and 35% of the estimated 24-h energy requirements, respectively (equal to basal energy expenditure calculated with the Harris Benedict equation times a physical activity level of 1.5). The breakfast was composed of one turkey breast sandwich with butter and of one yogurt with jam. The lunch was composed of one egg, cheese and butter sandwich, one plain yoghurt, and dried apple slices. At week 4 (end of run-in), all participants drank one 3.3 dL SSB (35 g sugar, 139 kcal) with each meal. Macronutrients and energy content of test meals are shown in Table 1. At week 16, they drank either SSBs or ASBs with meals according to the treatment arm. Overall energy intake was, therefore, higher for participants in the SSB arm (ca. 70% their 24-h energy requirements) than in the ASB arm (ca. 60%). Blood samples were collected every 120 min until 480 min after ingestion of the breakfast. 

### 2.3. Analytical Procedures

Plasma was immediately separated from blood cells by centrifugation at 1230× *g* for 10 min at 4 °C, and plasma aliquots were stored at −20 °C. Plasma metabolites (glucose, TG, non-esterified fatty acids (NEFAs), uric acid, and lactate) and urinary urea were measured by enzymatic methods (Randox Laboratories, Crumlin, UK). Insulin and glucagon were assessed by radioimmunoassay (Millipore, Billerica, MA, USA). Plasma fructose concentrations were measured by gas chromatography-mass spectrometry (GC-MS) as reported elsewhere [14].

IHCL content was measured by 1H-MRS on a clinical 3 Tesla MR system (TIM Trio, Siemens Medical, Germany) and VAT was determined using T1-weighted images of the abdomen, as previously described [15].

Total energy, carbohydrate, fat, and protein intakes were calculated from two-day food records with the Prodi 5.8 software (Nutri-Science Gmbh, Stuttgart, Germany). Each participant’s daily nutrient, energy, and beverage intakes were calculated by taking the average of two records obtained on two weekdays. Liquid sugar intake was calculated as the sum of sugars from SSBs, milk, coffee, tea, and alcoholic beverages. Sugars outside SSB were calculated by subtracting SSB sugars from the total sugar intake. 

### 2.4. Statistical Analysis

All values are expressed as means ± standard error of the mean (SEM). Data normality was checked with the Shapiro-Wilk test. Non-normally distributed data were log-transformed before statistical analysis. Baseline characteristics between ASB and SSB groups were assessed by Student’s unpaired *t*-tests. 

In a first calculation, we analyzed the global postprandial responses on the whole group of 26 subjects. For this purpose, we calculated, for each measured variable, the incremental area under the curve (iAUC) between time 0 min (i.e., immediately before breakfast) and time 480 min (i.e., 8 h after breakfast and 4 h after lunch); for these calculations, the mean of three fasting values obtained at time −30, −15, and 0 min were subtracted from postprandial values measured at time 120, 240, 360, and 480 min. When iAUC was significantly different from zero (which was the case for all variables except uric acid), a two-way ANOVA with interaction was performed to assess significant differences between the beginning and the end of intervention on the whole sample (effect of time), significant differences between intervention arms (intervention) and interactions between time and intervention.

In a second calculation, we performed a separate detailed kinetic analysis on all outcomes in the subgroups of 12 participants (three males, nine females, mean BMI 28.6 ± 1.1, age range 21–39 years) without hepatic steatosis (defined as IHCL < 5.5%), and in, a subgroup of 14 subjects (10 males, four females, mean BMI 32.8 ± 1.3, age range 20–43 years) with hepatic steatosis (IHCL > 5.5%). Comparisons were done by two-way ANOVA for repeated measures with interaction, with time (week 4 and week 16) and intervention (ASB or CTRL) as independent variables. Specific time-points with significant differences were identified with paired-t-tests when ANOVA showed a significant effect of intervention, or a significant interaction between time and intervention. 

All statistical calculations were performed with Stata 10 (Stata, College Station, TX, USA). *p* < 0.05 was considered statistically significant.

## 3. Results 

### 3.1. Anthropometric Variables, Dietary Intake, Fasting Plasma Metabolic Markers, and IHCL

Body weight, body composition, and fasting metabolic parameters at the end of the run-in period were already reported for all participants (*n* = 27) and did not differ significantly between groups. Fasting concentration of IHCL was somewhat larger in the CTRL group due to two subjects having IHCL > 325 mmol/L, however. The effects of intervention on spontaneous food intake have also been reported. Total energy intake decreased by 28%, and total sugar intake by 68% between week 4 and week 16 in the ASB arm, but did not change in CTRL. The effects of intervention on the main study outcome, IHCL, has been reported for all 27 participants [7]. IHCL decreased significantly in the ASB arm, but not in the CTRL arm, while body weight (90.1 vs. 91.0 in CTRL, 93.9 vs. 92.5 in ASB) did not change significantly in either group. There was also no statistically significant difference for total body fat mass, visceral fat volume, nor for all measured fasting metabolic substrate and hormone concentrations [7].

### 3.2. Postprandial Metabolic Responses

As a first step we analyzed postprandial time courses of each individual variables in all subjects. Ingestion of breakfast and lunch increased plasma glucose, fructose, lactate, triglyceride, uric acid, insulin, and glucagon concentrations, and decreased plasma NEFA concentrations. There was, however, no significant effect of time or intervention, and no significant time × intervention interaction. Data have not been displayed as figures for the whole group since they will be presented below in each subgroup. We then specifically assessed the postprandial effect of meal ingestion by reducing each variable to a single value, i.e., the incremental area under the curve (iAUC) cumulated between the beginning of breakfast to 4 h after ingestion of lunch and searching for effects of time (week 4 vs. week 16), treatment (CTRL vs. ASB), and time × treatment interaction (Table 2). This procedure assesses, globally, the responses of plasma metabolite concentrations induced by breakfast and lunch. Plasma glucose, insulin, lactate, and triglyceride concentrations increased after meals, resulting in large, positive iAUC, while plasma NEFA concentrations decreased, resulting in negative iAUCs. There was no significant effect of intervention, nor time × intervention interactions for any of these variables, but there was a trend for time effect (*p* = 0.06) and for a time × intervention interaction (*p* = 0.08) for lactate. Fructose and glucagon both increased slightly after meals, with a significant effect of intervention for glucagon and a significant time × intervention interaction for fructose, which decreased in the ASB arm and increased in the CTRL arm. iAUC for uric acid were not significantly different from zero, indicating no significant postprandial change.

We then ran the same analysis separately in the subgroups of participants without and with hepatic steatosis separately. Participants with hepatic steatosis had more cardio-metabolic risk factors, i.e., higher BMI, VAT, and Homeostasis Model Assessment of Insulin resistance (HOMA) index, plasma triglyceride and cholesterol concentrations, uric acid concentrations, and lower HDL-cholesterol concentrations. They also had larger decreases of IHCL with ASB [7].

**Figure 3:**
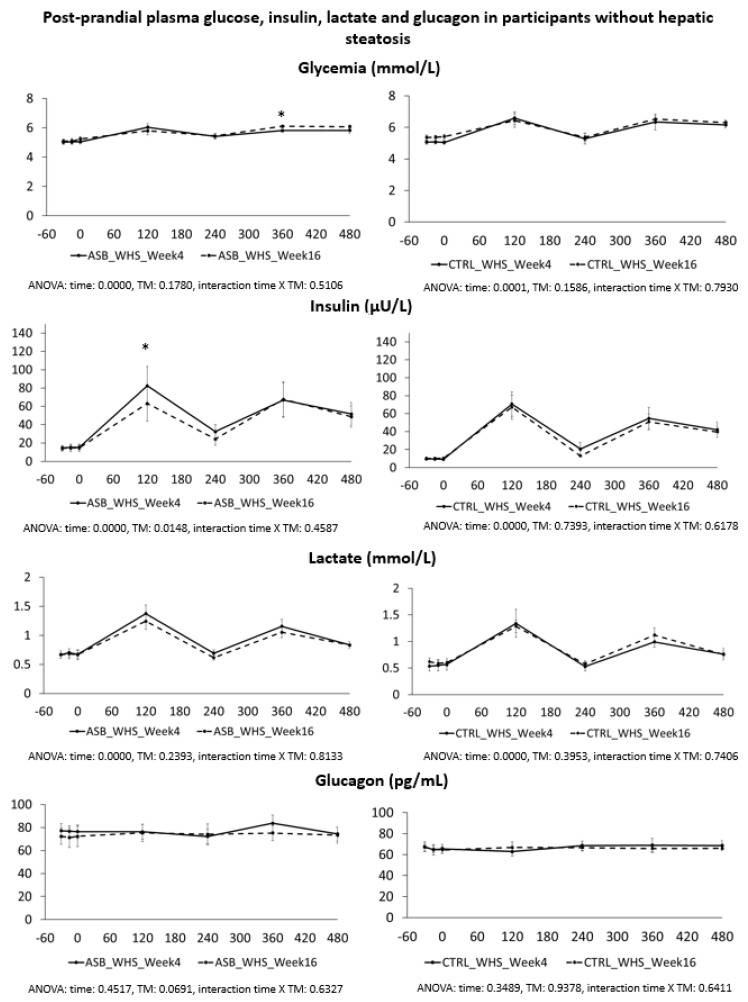
Plasma concentrations of glucose, insulin, lactate and glucagon in participants without hepatic steatosis. Data are expressed as mean ± SEM.; solid lines correspond to week 4, dotted lines to week 16. Results for ASB arm are shown on the left part, and for CTRL arm on the right part of the graph. Statistics (two-way ANOVA for repeated measurements with interaction) are reported at the bottom of each graph.

**Figure 4:**
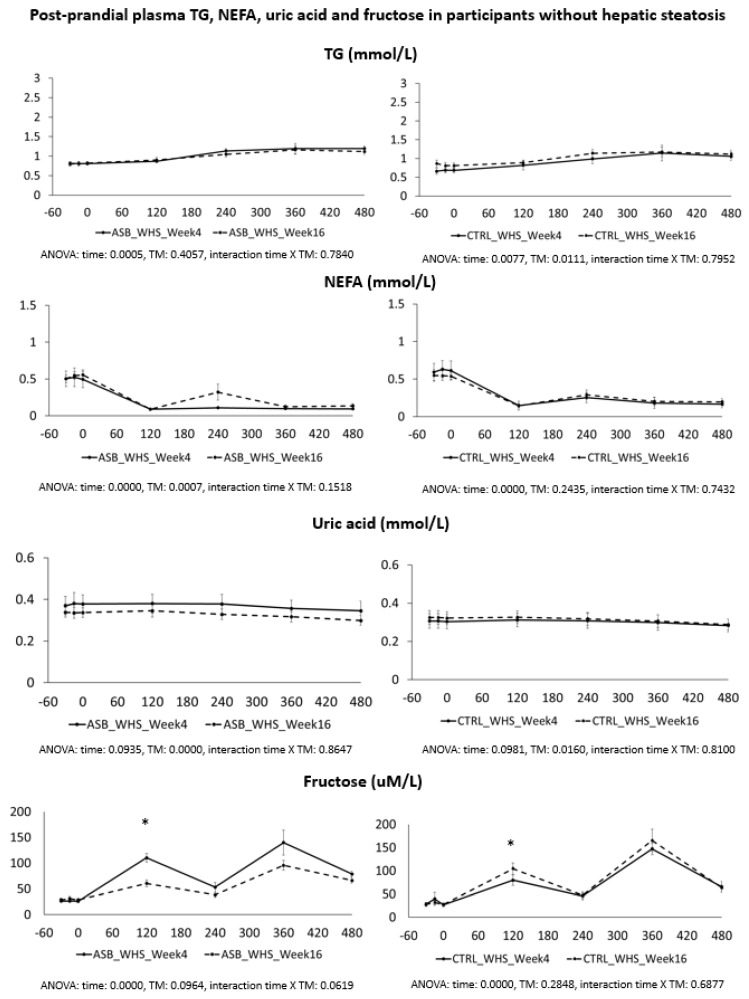
Plasma concentrations of TG, NEFA, uric acid, and fructose in participants without hepatic steatosis. Data are expressed as mean ± SEM.; solid lines correspond to week 4, dotted lines to week 16. Results for ASB arm are shown on the left part, and for CTRL arm on the right part of the graph. Statistics (two-way ANOVA for repeated measurements with interaction) are reported at the bottom of each graph.

Postprandial plasma glucose, insulin, lactate, and glucagon concentrations in participants without hepatic steatosis are shown in Figure 3. Peak plasma insulin and lactate concentrations after breakfast were significantly lower at week 16 than at week 4 in the ASB arm, but not in the CTRL arm. Postprandial plasma TG, NEFA, uric acid, and fructose are shown in Figure 4. Except for lower postprandial fructose concentrations after intervention in the ASB arm, these parameters were not significantly changed after intervention. 

**Figure 5:**
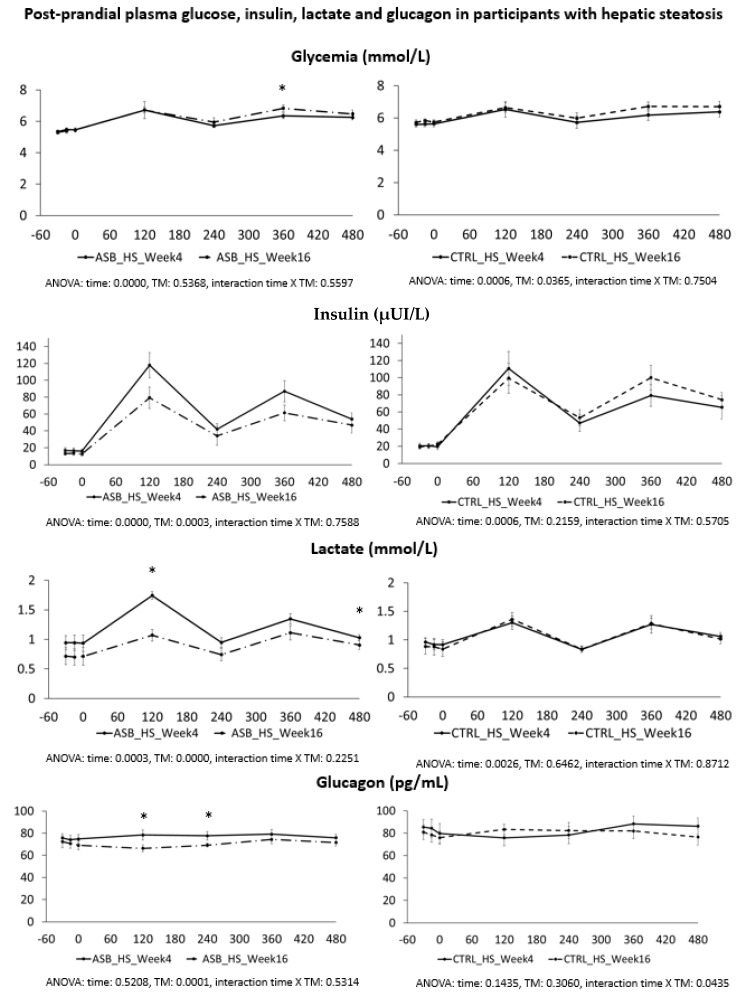
Plasma concentrations of glucose, insulin, lactate and glucagon in participants with hepatic steatosis. Data are expressed as mean ± SEM.; solid lines correspond to week 4, dotted lines to week 16. Results for ASB arm are shown on the left part, and for CTRL arm on the right part of the graph. Statistics (two-way ANOVA for repeated measurements with interaction) are reported at the bottom of each graph.

Postprandial plasma glucose, insulin, lactate, and glucagon concentrations in participants with hepatic steatosis are shown in Figure 5. Peak lactate concentrations after breakfast and lactate concentration at 480 min were significantly lower at week 16 than at week 4 in the ASB arm, but not in the CTRL arm. 

**Figure 6:**
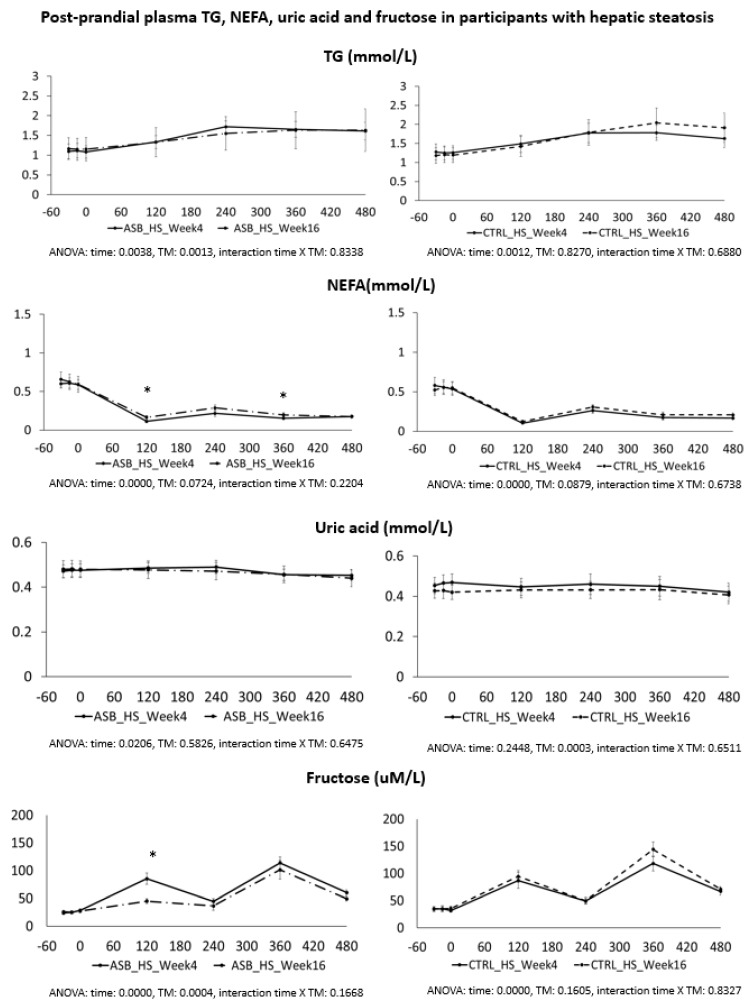
Plasma concentrations of TG, NEFA, uric acid, and fructose in participants with hepatic steatosis. Data are expressed as mean ± SEM.; solid lines correspond to week 4, dotted lines to week 16. Results for ASB arm are shown on the left part, and for CTRL arm on the right part of the graph. Statistics (two-way ANOVA for repeated measurements with interaction) are reported at the bottom of each graph.

Postprandial plasma TG, NEFA, uric acid, and fructose concentrations are shown in Figure 6, and were not statistically different at week 4 and at week 16, except for a slightly lesser suppression of NEFA and higher peak fructose concentrations in the ASB arm.

## 4. Discussion

The present worldwide epidemics of obesity and of related metabolic diseases are the consequence of an excess energy intake relative to energy expenditure, largely driven by environmental factors [16,17]. There is no question, based on the first law of thermodynamic, that obesity is associated with storage of large amounts of energy within body fat and, hence, this blunt statement holds true whether excess energy intake results of altered homeostatic mechanisms or of factors merely linked to modern lifestyle. Sugar-sweetened beverages and fruit juice consumption worldwide average 0.74 serving per day for the whole population. It shows some between-country variations, with an average consumption of 0.76 servings per day in high-income countries compared to 0.46 in low-income countries. Within each country, it also shows large inter-individual variations, mainly according to age and gender, with slightly higher values observed in males and in young adults [18].

SSBs, more than other energy-dense foods, have been proposed to be an ominous threat to body weight control and metabolic health for two main reasons. First, SSBs contain energy, yet their intake is triggered either by thirst, i.e., by physiological stimuli regulating body fluid homeostasis, or by social occasions. As such, SSB may escape the normal homeostatic mechanisms by which solid foods inhibit food intake [19]. Second, SSBs provide large amounts of mono- or disaccharides, of which approximately half is fructose. This monosaccharide first converts into glucose, lactate, or fat through the actions of specific enzymes located in splanchnic organs before being metabolized by extra-hepatic tissue [20]. There is, therefore, a concern that a high fructose intake, whether from SSBs or from “solid” foods, may impair blood glucose and lipid homeostasis by excessively stimulating gluconeogenesis and DNL even in the absence of excess energy intake. In support of this hypothesis, healthy volunteers had increased hepatic DNL and blood lipids when fed a high-fructose weight-maintenance diet [11,21] 

The REDUCS study was designed to assess the effects of SSBs reduction on IHCL, but not on energy intake. Furthermore, it was of too short duration to assess the effects on body weight. As already discussed elsewhere [7] it, however, decreased intrahepatic fat concentration, which may be an early marker of a negative energy balance. In addition to the measurement of IHCL as the primary study outcome, the experimental protocol included a detailed assessment of postprandial metabolism after ingestion of breakfast and lunch at the end of the run-in period, and again at the end of the intervention [7]. All participants had consumed their usual diet with SSBs during the run-in period, and received breakfasts and lunches, each ingesting one SSB together with both lunch and breakfast. Breakfast, lunch, and SSBs together accounted for about 70% of 24-h energy requirements, i.e., corresponded to the usual energy consumed with these meals in a typical European diet. After intervention, participants of the CTRL arm continued on their usual SSB-containing diet for 12 weeks, and received the same breakfast and lunch together with SSBs. In contrast, the participants in the ASB arm had consumed an SSB-reduced, possibly hypocaloric diet during the preceding 12 weeks, and received the same breakfasts and lunches as after run-in, i.e., providing ca. 60% of the calculated energy requirement and ASBs containing no calories. Comparing the metabolic profile in the ASB vs. CTRL groups at week 16, therefore, assesses both the effects of a chronic fructose-reduced diet vs. a chronic high fructose diet, and the acute effect of a fructose-reduced hypocaloric meals vs. a fructose-containing meal.

Based on the proposal that fructose has severe adverse metabolic effects of its own, i.e., independent of effects on energy balance or body composition, one would have expected significant alterations of postprandial metabolic parameters. The effects of intervention on fasting metabolic markers have already been reported for the 27 participants of the study [7], and were not different for the 26 subjects included in the present analysis. Our present data further support our initial report. Participants in the ASB group consumed, on average, 65% less total sugar than participants in the CTRL group over 12 weeks, but there was no significant difference in postprandial TG and uric acid concentrations. This is particularly striking since test-meals contained the same amount of carbohydrate, fat, and protein from “solid” foods in both arms, but an additional 120 kcal and 15 g fructose with SSB in CTRL arm only. The global effect of ASB on glucose homeostasis is most likely under-evaluated by our data since we only obtained blood samples 2 and 4 h after ingestion of breakfast and lunch, while ASB most likely led to maximal reductions of glucose, lactate, and insulin between 30 and 90 min after meal ingestion.

Based on several reports documenting the occurrence of fasting and postprandial hypertriglyceridemia in subjects fed controlled high-fructose diets [22,23] we had expected that reduction of SSBs’ consumption would come along with lower postprandial TG concentrations. In contrast with early postprandial glucose and insulin responses, plasma TG concentrations increase slowly over several hours [14,23], with a progressive increase between post-breakfast and post-lunch responses [22]. This pattern of TG response was, therefore, adequately assessed by collecting blood samples at two hours intervals. We, however, did not observe any significant decrease in the ASB arm, suggesting that neither the reduction of total sugar intake over the 12 previous weeks, nor the consumption of a single SSB together with meals, had much impact on postprandial TG concentrations. The discrepancy may be due the fact that supplementation studies [22,23] had used high, supraphysiological fructose doses. Our present data may simply indicate that consumption of one standard 3.3 dL SSB serving with a standard meal provides too low a fructose load to induce statistically and clinically relevant effects on these parameters.

We also expected that uric acid concentration would be decreased in the ASB arm due to reduced fructose intake. Fructose is well known to increase hepatic uric acid synthesis [24] and may decrease renal uric acid excretion [25,26]. As for TG, we did not observe any significant changes in uric acid concentration in the ASB arm. There are, however, many factors which significantly impact uric acid concentrations, including genetic factors, dietary factors, or alcohol intake. One may, therefore, hypothesize that fructose contribution to uric acid concentrations was relatively small. Alternatively, it is possible that SSB reduction induced a negative energy balance which did not significantly impact on body weight due to the short duration of intervention, and that a reduction in uric acid from SSB reduction was balanced by an increased uric acid release from weight loss in this group. A recent study also reported no effects of replacing SSBs with pure glucose- or pure fructose-drinks during four weeks in Hispanic American adolescents with hepatic steatosis. Plasma TG concentration did not increase, and were even non-significantly decreased by ca. 30%, when SSBs were replaced by pure fructose drinks. In contrast, there was a significant reduction of plasma C-reactive proteins and oxidized low-density lipoprotein when SSBs were replaced by glucose, but not fructose, drinks, still suggesting that fructose was associated with adverse metabolic effects [27]. In contrast, another recent study reported significant reductions of fasting and postprandial plasma glucose insulin and triglyceride concentrations when added sugars were replaced with isocaloric amounts of starch over nine days in Hispanic and African American children [28]. This was a non-controlled study, however, and participants lost, on average, 1 kg body weight over the one-week intervention. It is, therefore, highly likely that these effects were mainly due to hypocaloric feeding; this rapid weight loss was also likely of origin of the increase in plasma uric acid concentration reported in this study [28]. 

We also hypothesized that fructose may exert more deleterious metabolic effects in insulin-resistant, than insulin-sensitive, subjects. In this regard, it is of interest that the study participants with hepatic steatosis were insulin resistant, but had higher plasma TG and uric acid concentrations than those without hepatic steatosis. Even in this subgroup, however, postprandial plasma TG and uric acid did not decrease after 12 weeks of intervention with sugar/fructose restriction. This suggests that factors others than the mere intake of SSBs play a major role in their hypertriglyceridemia and hyperuricemia. One may argue that the aforementioned studies were of too short a duration to observe the full range of beneficial effects of SSB reduction. However, while many intervention studies in which fructose or sugar were added to the usual daily intakes of participants documented significant increases in plasma triglycerides [29,30], few interventions involving the reduction of dietary sugar over several months or years have reported on plasma lipids. One such study, which had previously reported a lower BMI increase in children receiving ASBs than in controls receiving SSBs [31], has recently reported published data related to the study’s secondary outcomes: in spite of a two-year intervention, there was also absolutely no effect of replacing SSBs with ASBs on fasting blood triglyceride concentrations [32].

SSB reduction had no significant effect on postprandial metabolic risk factors, yet significantly reduced IHCL, which was the primary endpoint of the study. Due to its two-step metabolism, which includes a conversion of its carbons into glucose, lactate, and fatty acids, fructose affects muscle and adipose metabolism through mechanisms distinct from glucose or dietary fat. There is growing evidence that subtle alterations of inter-organ substrate fluxes, leading to ectopic accumulation of fat in the liver, muscle, and possibly other tissues, may be responsible for its long-term adverse metabolic effects.

Our study has several weaknesses, however. First, it included a small number of subjects and, hence, may not have the statistical power required to detect small differences. To compensate for this weakness, we however performed an 8-h metabolic monitoring with multiple measurements in very well controlled conditions. Second, it was an outpatient study, with no direct assessment of adherence to intervention. Every participant received every other week her/his estimated number of beverage cans needed, and adherence assessed from counting the empty cans returned at the following visit was highly satisfactory, however. Furthermore, we observed drastic decreases in 24-h urinary fructose excretion after intervention, supports the hypothesis that adherence was high. Third, we assessed the intake of solid foods and physical activity with methods known to have low sensitivity and, hence, cannot estimate how much of the SSBs’ energy was compensated for by increased solid food consumption or decreased physical activity in the ASB group. Fourth, we have not included an intervention arm with water replacing SSBs and, hence, cannot assess whether artificial sweeteners exerted direct metabolic effects.

## 5. Conclusions

The present results indicate that a 12-week replacement of SSBs with ASBs failed to significantly change postprandial plasma triglyceride and uric acid concentrations in overweight subjects. This observation remained valid when the analysis was restricted to a subgroup of participants with hepatic steatosis. Although they may appear as unexpected, these results are consistent with several other published studies. The overall interpretation and practical implications of this study, and of the scientific literature on sucrose and health at large, may appear somewhat confusing. We believe that sucrose, given its high consumption in most of the world on one hand, and its hedonic properties on the other hand, is very likely to be an important contributor to the high prevalence of obesity. We also believe that SSBs, whose consumption is dependent on thirst and social occasions, rather than hunger, are particularly problematic. However, the hypothesis that fructose, per se, has adverse metabolic effects even when consumed as part of an energy-balanced diet may be somewhat exaggerated. Few intervention studies, having used large doses of fructose-containing sugars, actually support it. Our present results instead show that the postprandial metabolic profile was not markedly different when participants reduced their usual SSB consumption. Based on studies like this one, and on other reports showing no dramatic improvement of cardio-metabolic markers after SSBs reduction, one may have to reconsider the proposal that SSB reduction will be efficient to prevent or revert obesity unless associated with other drastic public health interventions. Public health interventions on SSBs, although entirely appropriate, should, therefore, not distract us from other, energy, fat, or salt-rich foods. 

## Figures and Tables

**Table 1:** Nutrient content of the test meals.

	Week 4 Test Meals	Week 16 Test Meals	
		ASB	CTRL
Breakfast			
Energy (kcal)	748	658	762
Starch (g)	50	53	51
Sucrose (g)	49	15	49
Fat (g)	20	22	21
Protein (g)	23	25	23
Lunch			
Energy (kcal)	981	895	1009
Starch (g)	67	71	70
Sucrose (g)	56	22	57
Fat (g)	28	30	29
Protein (g)	31	33	32

**Table 2:** Postprandial incremental area under the curve (iAUCpp) of metabolites and hormones concentrations in all participants. Values are expressed as mean ± 1 SEM.

	ASB		CTRL		*p* Values (2-Way ANOVA)		
	(*n* = 7 ♂, 6 ♀)		(*n* = 6 ♂, 7 ♀)				
	Week 4	Week 16	Week 4	Week 16	Time	Intervention	Time × Intervention
Lactate iAUCpp (mmol/L × 480 min)	200.1 ± 23.2	153.0 ± 24.7	161.3 ± 38.7	175.6 ± 33.8	0.3503	0.0586	0.0872
Glucose iAUCpp (mmol/L × 480 min)	645.3 ± 27.6	700.6 ± 62.7	644.2 ± 112.7	645.1 ± 69.4	0.5045	0.5034	0.5192
NEFA iAUCpp (mmol/L × 480 min)	−144.3 ± 22.2	−129.0 ± 20.5	−132.9 ± 31.4	−106.4 ± 28.4	0.2240	0.8856	0.7383
Glucagon iAUCpp (pg/mL × 480 min)	5291.1 ± 1052.0	4684.6 ± 855.8	4367.1 ± 659.5	5248.2 ± 1152.2	0.8551	0.0148	0.3273
Insulin iAUCpp (μU/mL × 480 min)	23,502.9 ± 3246.0	17,734.2 ± 2772.0	21,216.0 ± 4436.4	21,348.0 ± 4123.9	0.1380	0.2571	0.1213
Triglycerides iAUCpp (mmol/L × 480 min)	218.9 ± 23.7	189.5 ± 47.2	214.2 ± 46.7	240.1 ± 67.0	0.9506	0.6001	0.3338
Fructose iAUCpp (μM/L × 480 min)	24.0 ± 2.1	19.12.4	19.5 ± 4.1	21.3 ± 5.0	0.2231	0.6329	0.0456
Uric acid iAUCpp (mmol/L × 480 min)	27.1 ± 25.9	16.2 ± 11.2	22.6 ± 39.8	28.5 ± 35.9	NA ^1^	NA ^1^	NA ^1^

^1^ NA: not applicable: ANOVA was not performed because iAUCpp were not significantly different from 0.
